# Progress in Medicine

**Published:** 1899-04-01

**Authors:** 


### Progress in Medicine.

### FEVERS.

**Influenza.**—The revival of this epidemic in the dry weather following the long winter rains has brought forward at least one suggestion for treatment. J. Carne Ross,[1] who advocated the antiseptic value of a decoction of cinnamon in scarlatina some time ago, has recommended the same drug for influenza. He uses half-an-ounce of the decoction, or two of Burroughs and Wellcome's tabloids every half-hour for two hours, and then every hour till the temperature is normal; afterwards it is given four times a day for four days. The treatment must be commenced within 24 hours of the onset of the disease, and the writer has found an entire absence of complications, and very rapid recovery in all cases so treated. He insists on patients remaining indoors for 24 hours after the temperature is normal. The *Lancet*[2] refers to the fact that this is the tenth year of the epidemic which had been absent from us previously for 43 years. Sisley's book shows that it had visited this country before that on the average every 15 years for the last three centuries, and numerous notices of its attacks in still earlier times are to be found. The extraordinary variety of its symptoms and complications leads to great difficulty in its diagnosis, which would be lessened if the identification of Pfeiffer's bacilli were more easy. Wynckoop holds that they can be readily made out in the sputa by anyone who is familiar with their special form. The two great dangers are from pneumonia and nerve complications, the latter sometimes appearing years after the disease has run its course. The respiratory tract being the place of entrance, dwellers in close, ill-ventilated houses are most readily attacked, and its effects are most to be dreaded in the aged, the over-worked, alcoholics, and invalids.

**Small-pox.**—The working of the new Vaccination Act has led to much discussion of its obligations. The *British Medical Journal*[3], in referring to the solicitations of parents who insist on only one or two marks, says that the only answer should be either thorough vaccination, or a recourse to the conscience clause. No medical man should by inefficient vaccination increase the great number of half-protected children who figure so largely in every epidemic, since the parent is able to take the responsibility on himself by a declaration.

It might be added that efficiency depends rather on the number of vesicles than of punctures; a larger number may be formed on a linear scratch than on four simple stabs.

The *Lancet*[4] points out that glycerinated lymph may vary widely in its purity. Spore-bearing microbes cannot, as a rule, be destroyed by the glycerine, and some makes of lymph are not free even from the non-sporulating kinds, for it is not merely mixing the lymph with glycerine, but the long-continued action of glycerine under certain conditions which purifies it. We must hope that the use of purer lymph and of antiseptic precautions under the new Act will result in preventing those occasional accidents which have been a cause of so much dislike to vaccination. About a third of the children born last year escaped protection, and indeed a fourth of the Boards of Guardians had evaded the old law, so that the results of this "tremendous experiment" in legislation for increasing the popularity of vaccination will be awaited anxiously. No re-vaccination Bill is, it seems, to be brought in this session. Stanley Kent[5] has succeeded in growing the microbe of vaccinia in milk, and in a mixture of albumen, glycerine, and water. He describes it as a diplobacillus, which stains with some difficulty, and is found in the vesicles and surrounding tissues. Pure cultures when inoculated caused typical new vesicles containing the organism in large numbers, and the animals were afterwards resistant to vaccination with ordinary lymph.

**Typhoid.**—Very few new suggestions have of late appeared for treatment. Andriewsky[6] has tested calomel in four-grain doses three times a day on 71 patients. This was continued until the evening temperature fell to normal. Stematitis never occurred, nor was the diarrhœa aggravated, while the disease ran a mild course, and often came to an end prematurely. The mortality was very low (2·8 per cent.), and the writer considers that, though not a specific, calomel is a most useful remedy in typhoid. The cold bath treatment has been supported very ably by the published results of F. E. Hare,[7] of Brisbane, who shows that the case mortality of the disease is by it reduced nearly 50 per cent.; that nearly all the distressing symptoms due to pyrexia, and many of those arising otherwise, are alleviated better by baths than by any other treatment; that nutrition is maintained and recovery quickened; and that even in fatal cases life is prolonged. Indeed, the increased feeling of comfort and refreshing sleep is so marked as to lead many patients to beg for a continuance of the baths during convalescence. The difficulties of diagnosis are increased by the existence of fevers produced by the Malta micrococcus and the bacillus enteritidis of Gärtner.

Durham[8] shows that outbreaks of the latter class have been traced to the consumption of insufficiently cooked meat of diseased animals. The same bacillus has been conveyed by parrots, and the resulting fever has been termed psittacosis. The symptoms are very variable; sometimes there is severe diarrhœa, with collapse ending fatally in a few days, perhaps accompanied with hæmatemesis and melæna. Other cases of a milder type appear, but recovery is slow and tedious. The incubation period in the meat epidemics is very short, often only a few hours; while in the parrot disease this may extend to twelve days. Here, too, diarrhœa is less common, and the temperature often comes down suddenly, whereas in infection from unsound meat there may be relapses and irregular pyrexia. Some few cases are exceedingly difficult to diagnose clinically from true typhoid apart from the history of several persons being attacked after a common meal, or the presence of diseased parrots, and the Widal reaction itself is given by Gärtner's bacillus according to some writers. Whitaker[9] refers to typhoid without the usual anatomical lesions. In five cases diagnosed as typhoid which gave the serum test the usual lesions in the ileum were absent, but investigation showed the presence of the bacillus in the gall bladder, spleen, kidneys, or other organs. Similar cases have been recorded by Flexner, and show that typhoid infection may occur without the ordinary localisation. In no case where the bacilli have been found in the gall bladder has the serum test been negative. The serum test, indeed, in combination with the Diazo reaction is usually conclusive, even in obscure cases. The Diazo change is absent in the first few days, and after the twentieth day, and appears not only in typhoid between those dates but also in measles, phthisis of a severe type, and other tuberculous affections, but its chief defect is its occasional absence in true typhoid. However, Michaelis,[10] at the Verein für Innere Medizin, in Berlin, stated that he had been working at the Diazo reaction for seven years and never found it in health. He thinks it always appears in typhoids, and at an early stage, and lasts five to eight days, thus preceding the spots and enlargement of the spleen. If it remains a long time the disease will probably take a grave form. In measles such a continuance generally indicates pneumonia. When the reaction is found in erysipelas, pneumonic diphtheria, and still more in phthisis, it indicates a severe attack, and is independent of the presence or absence of fever. The prognosis in phthisis, where it is found, is very grave. He instanced cases where it was met with in apparent health and rheumatic fever, and here phthisis was found to be in course of development, though physical signs were at first absent. In chronic non-infective diseases it is never seen. Baginsky and others agreed as to the importance of the test, both for prognosis and diagnosis. J. C. Wilson[11] reports a case of typhoid where Widal's test was present, with pyrexia and headache, but no spots or splenic enlargement. A large quantity of albumen and casts were noted and disappeared. In six weeks' time a typical relapse, with all the symptoms of typhoid and a return of the albumen and blood, showed the correctness of the diagnosis of typhoid where the chief stress had fallen at first on the kidneys. A. E. Sansom[12] refers to the difficulty of distinguishing typhoid in some cases from influenza of a gastric type. In the latter we may get patchy broncho-pneumonia instead of the bronchitis of typhoid, and very doubtful spots, which may lie in an intercostal space like herpes, instead of the true typhoid rash. There may also be diarrhœa and a temperature, or melæna from purpura in influenza, which would increase the difficulty. Again, acute colitis may at first be indistinguishable, and some cases have been diagnosed as typhoid, but all the lesions have been found post-mortem confined to the colon.

The cardiac signs in typhoid show remarkable enfeeblement of the heart muscle, analogous to the curious weakness of the systemic muscles which the patient notices almost before anything else. Thus the pulse is soft and dicrotic. There may be mitral regurgitation from failure of the papillary muscles, and in the later stages the area of cardiac dulness is unusually small, because the heart has wasted just as the body muscles do; even the fœtal tic-tac sound may be heard at times, replacing the normal one. Yet all these changes pass away during convalescence, and Sansom doubts whether the sudden deaths ascribed to this form of myocarditis are not really due after all to poisoning of the higher nervous centres. Endocarditis is probably never produced by typhoid.

Among other difficulties of diagnosis we may refer to the peculiar degree of agglutinability of some breeds of the bacillus. McWeeney[13] reports that a strain which he cultivated from the bile of a certain case was most resistant to typical serums. While a ten per cent. dilution instantly clumped ordinary bacilli, it had not cleared the field of these hardy specimens in half-an-hour. Similar results were obtained with greater dilutions. The possibility of such peculiarities must therefore be accepted as increasing the perplexities of the Widal reaction. The writer also showed that the reaction normally depends on the effects of two substances, one contained in the serum and the other in the bacilli, that the serum need not be that of immunity, and that the phenomenon is not mere precipitation, and can take place with non-motile bodies, so that certain serums cause the agglutinisation of red blood corpuscles.

[1] Brit. Med. Jour., Mar. 18. [2] Lancet, Mar. 18. [3] Brit. Med. Jour., Feb. 4. [4] Lancet, Dec. 31 and Jan. 7. [5] Lancet, Dec. 17. [6] Brit. Med. Jour., Jan. 7. [7] Brit. Med. Jour., Dec. 17. [8] Brit. Med. Jour., Dec. 17. [9] New York Med. Jour., Jan. 14. [10] Glasgow Med. Jour., Mar. [11] Brit. Med. Jour., Feb. 4. [12] Clinic. Jour., Dec. 21. [13] Brit. Med. Jour., Feb. 11.

